# Acute affective responses to prescribed and self-selected exercise sessions in adolescent girls: an observational study

**DOI:** 10.1186/2052-1847-6-35

**Published:** 2014-09-25

**Authors:** Charlotte C Hamlyn-Williams, Paul Freeman, Gaynor Parfitt

**Affiliations:** 1 General and Adolescent Paediatrics Unit, Population, Policy and Practice, UCL Institute of Child Health, 30 Guilford Street, London WC1N 1EH, UK; 2 Sport and Health Sciences, College of Life and Environmental Sciences, University of Exeter, Exeter, UK; 3 School of Health Sciences, Sansom Institute for Health Research, University of South Australia, Adelaide, SA, Australia

**Keywords:** Affect, Physical activity, Exercise psychology, Autonomy, Adolescent exercise

## Abstract

**Background:**

Positive affective responses can lead to improved adherence to exercise. This study sought to examine the affective responses and exercise intensity of self-selected exercise in adolescent girls.

**Methods:**

An observational study where twenty seven females (Age *M* = 14.6 ± 0.8 years) completed three 20-minute exercise sessions (2 self-selected and 1 prescribed intensity) and a graded exercise test. The intensity of the prescribed session was matched to the first self-selected session. Intensity, affective responses and ratings of perceived exertion were recorded throughout the sessions and differences examined. Repeated measures ANOVAs were conducted to examine differences.

**Results:**

There were no significant differences in intensity between the prescribed and self-selected sessions, but affective responses were significantly more positive (*p* < .01) during the self-selected session. Ratings of perceived exertion were significantly lower (*p* < .01) during the self-selected session than the prescribed session. On average participants worked at 72% $\dot{V}O_{2}$ peak; well within the intensity recommended by the American College of Sports Medicine.

**Conclusion:**

Even though the intensity did not differ between the self-selected and prescribed sessions, there was a significant impact on affective responses, with more positive affective responses being elicited in the self-selected session. This highlights the importance of autonomy and self-paced exercise for affective responses and may have potential long-term implications for adherence.

## Background

The identification of exercise or physical activity (PA) that adolescents enjoy and experience as positive is important [1,2]. This is particularly important for females given the decline in PA that occurs as individuals enter adolescence [1,3]. It is vital to understand how an individual experiences exercise, as previous research [4] has indicated that the exercise experience can influence future PA behavior. The experience of positive affect during exercise impacts upon motivation and behavior with recent research confirming that affect may be the first link in the exercise adherence chain [5,6].

Research has explored whether allowing individuals to self-select their own exercise intensity compared to an external person prescribing an intensity elicits more positive affective responses [7-9]. Results from studies by Vazou-Ekkekakis and Ekkekakis [10] and Lind et al. [7] showed that affect was more negative when the intensity of exercise was prescribed by an external person, rather than chosen by the individual; the authors suggested that this decline in affect may be as a result of loss of autonomy. With adolescents, Sheppard and Parfitt [11] showed that during a 15-minute cycle exercise session affective responses became less positive over time in a prescribed, high intensity session, than in a self-selected session where affective responses remained stable and more positive. In a follow-up study, Stych and Parfitt [12] explored both quantitative and qualitative responses to self-selected exercise and found that low-active adolescents who reported feeling in control of their intensity and affective responses were more positive during the self-selected exercise sessions, than in the prescribed sessions.

While the majority of studies that have allowed participants to self-select intensity result in an exercise intensity within the levels recommended by the American College of Sports Medicine (ACSM) [13,14] exercise professionals typically prescribe exercise intensities rather than allow clients to self-select the intensity. This is presumably to ensure that all individuals are working at an intensity that would be beneficial to their health and to avoid over-exertion. Indeed, the ACSM exercise prescription guidelines have previously recommended exercise levels that maximize effectiveness and minimize risk [15]. However, Ekkekakis et al. [16] and the most recent ACSM standpoint [17] suggest that incorporating preference and a self-selected ‘comfortable’ exercise intensity will result in exercise that is pleasurable, safe, and effective. Indeed, for decades researchers have been promoting the importance of preference and pleasure in the exercise environment to support exercise motivation [18,19], with qualitative data from both adults [20,21] and adolescents [12] indicating that ‘choice’ and ‘control’ contribute to positive affective responses. These data are consistent with Self-Determination Theory (SDT) [22].

One focus of SDT is the motivational implications of self-selected (autonomous) and prescribed or dictated (non-autonomous) behaviors. Deci and Ryan [22] suggested that ‘autonomy’ refers to the degree of freedom that an individual perceives to have to perform the behavior of his or her own choice. The SDT suggests that the degree of pleasure that an individual experiences when they act autonomously is likely to be higher than that experienced when behavior is externally controlled. Deci and Ryan [22,23] therefore suggested that under autonomous conditions, positive affect is more likely to occur.

Studies with adults have sought to examine whether manipulating an exerciser’s perception of autonomy, by allowing or disallowing an individual to set his/her own exercise pace, could have an impact on affective responses [8,20]. Vazou-Ekkekakis and Ekkekakis’ [8] results were consistent with SDT and indicated that the loss of perceived autonomy in setting the level of exercise intensity negatively impacted participants’ affective responses and enjoyment. However, a limitation acknowledged in this study was the fixed order methodology (all participants completed the self-selected condition first). Given that Rose and Parfitt [20] recorded an order effect, where active women experienced more positive affect during exercise and greater competence than sedentary women when the self-selected condition was completed first, future research needs to counterbalance the prescribed and self-selected session to control for this effect. Further, Williams and Raynor [24] recently examined the independent effects of PA intensity and choice of intensity and compared affective responses to, and preference for, yolked-self-selected intensity versus a prescribed, imposed higher intensity session. They found that low-active women preferred leisure PA (walking) that was of a self-selected intensity compared to PA that was imposed or prescribed at a higher intensity. However, no significant difference was found in core affective response between the self-selected and prescribed sessions.

In addition to affective responses, previous research has demonstrated different perceptual ratings of effort for specific exercise intensities when the process has differed between active and passive control. For example, in an effort production protocol, in which the individual had to actively regulate the exercise intensity (such as in a self-selected session), the perceptual responses were lower than when the individual was passively estimating the effort [25].

The primary objective of this study was to examine exercise-related affective responses in adolescent females (a population where levels of PA rapidly decline) when autonomy to modify exercise intensity was manipulated with the use of a self-selected protocol and prescribed (but matched to self-selected) paradigm.

We hypothesized that affective responses would be more positive during the self-selected session than during the prescribed session and ratings of perceived exertion (RPE) would be lower during the self-selected session than during the prescribed session. Descriptive data was also analyzed to identify whether, on average, the self-selected exercise intensity would be within the recommended range of the ACSM; 50-85% $\dot{V}O_{2}$ max/peak [15].

## Methods

### Participants

Twenty seven adolescent females (age *M* = 14.6 years ± 0.8 years; mass *M* = 54.7 kg ± 9.1 kg; height *M* = 1.6 m ± 0.1 m; BMI *M* = 20.9 kg/m^2^ ± 3.0 kg/m^2^) from schools across South West England were recruited into the study from a convenience sample of interested schools, contacted by telephone, post or email. Power calculations using GPower [26] indicated that a sample size of 26 participants was required for this study (α = 0.05, power =0.95).

Information letters were sent to school PE staff to assess interest levels. Information sheets and consent forms were then sent to parents and children. Participants were all Caucasian, healthy and free from muscular-skeletal injury.

### Ethics

For some unfit individuals it was acknowledged that the exercise sessions and maximal physical activity conditions may result in some discomfort related to their exertion. However, the associated risks were low, and were further reduced by careful explanation of the test procedures by properly trained staff. A qualified first aider was available through the testing procedures. All participants were free to terminate the testing at any stage, and testing was terminated if participants displayed unusual signs of discomfort. Participants were able to withdraw from the study at any time without having to give a reason and without any disadvantage to them.

All information obtained was stored on a computer in coded form, anonymised and individual results were confidential to the participant and the research team. Participants and their parents were informed that the results of the study may be published but that any data included would in no way be linked to any specific participant.

As the study involved participants under 18 years of age, all personnel directly involved with the project testing were CRB checked (enhanced level) and a member of school PE staff was present during all exercise sessions.

### Measures

#### Affect

Affective valence (the pleasure/displeasure one feels) was measured using the Feeling Scale (FS) [27]. Participants rated their current level of affective valence on an 11-point bipolar scale ranging from +5 to −5, with verbal anchors of *very good* (+5), *good* (+3), *fairly good* (+1), *neutral* (0), *fairly bad* (−1), *bad* (−3) and *very bad* (−5). The FS has been previously validated [28,29] and successfully used with adolescents [30-33].

#### Ratings of Perceived Exertion (RPE)

Perceived exertion was assessed using the Eston-Parfitt (E-P) curvilinear Ratings of Perceived Exertion Scale [34]. This scale depicts a character at various stages of exertion on a concave slope with a progressively increasing gradient at the higher intensities, with anchors between 0 and 10, ranging from *very, very easy (0), easy (2), starting to get hard (4), very hard (7)* to *So hard I am going to stop (10)*. Previous research has confirmed the robustness of the E-P Scale [34]. The same verbal instructions were given to all participants prior to undertaking any exercise, and participants were given several minutes to familiarize themselves with the scale. For full instructions see [34].

#### Instruments

##### K4 breath analyzer

The K4 Cosmed Breath analyzer (Cosmed K4, Italy), with a junior face mask, head net and harness, was used in order to measure breath by breath expired gases throughout the duration of the testing. The K4 has been shown to provide valid measurements of oxygen uptake across a range of exercise intensities [35]. The K4 was calibrated before every test in accordance with manufacturer’s guidelines against known concentrations of cylinder gases and a 3-L syringe (for flow volume).

##### GENEActiv physical activity monitor

To understand the habitual PA levels of the sample, participants wore a GENEActiv accelerometer on their dominant wrist for seven days following the completion of all of the exercise sessions. The accelerometer was set to record at 100Hz. The GENEActiv has been previously validated as an accurate and reliable measure of children’s activity [36]. Data were analyzed to determine the total time spent doing PA, which was treated as a descriptive factor in the study. Participants’ data were used if they had recorded ≥10 hours/day of wear time for at least three week days and one weekend day [37].

### Procedures

Prior to the study participants, as well as their parent, guardian or care giver, read and signed informed assent and consent forms approved by the ethics committee at the University of Exeter. Height and mass were measured upon arrival at the gym and body mass index (BMI) was calculated.

Participants took part in four gym-based exercise sessions at least 48-hours apart: three sub-maximal sessions and one graded exercise test. The initial sub-maximal session was a self-selected, familiarization session. This was followed by self-selected and prescribed exercise sessions, which were randomized and counterbalanced to control for any order effects that may have occurred if all participants had performed the second self-selected session and prescribed exercise sessions in the same order. The final session was a maximal graded exercise test (GXT). Following completion of all of the exercise sessions, PA levels were measured using the GENEActiv accelerometer for the subsequent seven days.

#### Session 1 – self-selected familiarization session

Session 1 acted as a familiarization session for the participants to ensure that they were comfortable with the sensations associated with running and walking on a treadmill, as well as giving them the opportunity to familiarize themselves with the FS and E-P scale. Following a 3-minute warm-up at a self-selected comfortable speed on the treadmill, each participant was asked to ‘*select a comfortable pace of any intensity you want, it can be a walk or a run, whatever feels the most comfortable*.’ Participants were also told they could modify the self-selected intensity at any point throughout the 20-minute exercise session, but the time and speed of the session was kept blind from participants. Any adjustments to the intensity were recorded.

#### Session S – self-selected

Session S followed an identical protocol to the first self-selected exercise session.

#### Session P – prescribed

Participants again warmed up for 3-minutes at a comfortable speed on the treadmill and then a prescribed exercise intensity was set. The prescribed exercise session was set to the same speed and intensity as the initial self-selected exercise session and any modifications to the speed that the participants made in the first session were replicated by the investigator. Time and speed were again kept blind from participants so as not to contribute to affective responses or RPE but they were told when the speed would be changing and whether it would be increased or decreased.

During each session (including the familiarization session) direct measurements of oxygen uptake were collected and analyzed using the K4 portable online gas analyzer. Responses to the FS were taken in the first minute of the session (min 1) as well as the last 45 s of each 5-minute period (minutes 5, 10, 15 and 20). Any changes in intensity were recorded throughout the 20-minute exercise sessions. RPE was recorded at the same time points as the FS.

#### Session 4 – graded exercise test

In the final visit to the gym, all participants completed a maximal graded exercise test (GXT) to volitional exhaustion to establish peak aerobic capacity $\left(\dot{V}O_{2\mathrm{peak}}\right)$ and Ventilatory Threshold $\left(\dot{V}T\right)$. The GXT was completed on a Fitness Suite Treadmill (TechnoGym Run Race Treadmill) in 1-minute stages at a comfortable, self-selected running speed, increasing the gradient by 1% every minute after a 3-minute warm-up. The test was continued until the participant reached volitional exhaustion. Expired gases were measured using the K4 breath analyzer. At the end of every incremental step during the test (last 20-seconds of every period) FS and RPE scores were collected.

### Data analysis

Ventilatory Threshold was calculated for all participants. VT was determined from the GXT data as the first disproportionate increase in carbon dioxide output $\left(\dot{V}CO_{2}\right)$ relative to $\dot{V}O_{2}$. This was achieved from visual inspection of individual plots of $\dot{V}CO_{2}$ versus $\dot{V}O_{2}$ by two independent assessors. Breath-by-breath data from each test were smoothed to average every 10 seconds to make visual identification of the break point clearer [38].

A 2 (condition: self-selected or prescribed) by 5 (time: min 1, 5, 10, 15 and 20) repeated measures ANOVA on $\%\pm \dot{V}O_{2}‒\mathrm{at}‒\mathrm{VT}$ was conducted to determine any differences in intensity between the prescribed and self-selected sessions. Further, the $\%\dot{V}O_{2}$ peak at minute 1, 5, 10, 15 and 20 of the self-selected exercise session was examined to determine whether the intensity was within the ACSM recommended levels.

Mixed model (condition by time by order) ANOVAs were conducted on affective responses and RPE to explore differences between the self-selected and prescribed exercise sessions across time points (min 1, 5, 10, 15, 20) as repeated measures factors and order (prescribed first or second) as a between-subjects factor.

All statistical analyses were conducted in SPSS (SPSS 19.0; SPSS, Chicago, IL). Where sphericity was violated, Greenhouse-Geisser was used to adjust the degrees of freedom and these are reported. Partial eta squared is also reported as a measure of effect size.

## Results

Descriptive anthropometric (height, mass and body mass index (BMI)), physiological (ventilatory threshold and VO_2_ peak), and PA data (minutes spent doing moderate to vigorous physical activity (MVPA)) are displayed in Table 1. Results show that, based on cut points proposed by Phillips et al. [36] participants on average took part in 285 (±114.2) minutes of MVPA/week. Four out of the 28 participants exceeded the recommended 60 minutes of PA/day.

**Table 1:** Mean descriptive characteristics of participants

	**Mean**	**Standard deviation**
Height (m)	1.6	0.06
Mass (kg)	54.8	9.1
BMI (kg/m^2^)	20.9	3.0
$\dot{V}O_{2}$ Peak (ml.min^−1^.kg^−1^)	42.9	7.7
Ventilatory Threshold $\left(\dot{V}T\right)$ (ml.min^−1^.kg^−1^)	33.5	5.8
MVPA per day (mins)	41.0	16.4
MVPA per week (mins)	285.0	114.2

During the self-selected intensity session, participants on average made 5.2 (±2.9) adjustments to the intensity, predominantly within the first 10-minutes of the session. Speeds ranged from 3.5 km/h to 11.5 km/h, with 68% of changes resulting in an increase in speed, and 32% resulting in a decrease in speed.

**Figure 1:**
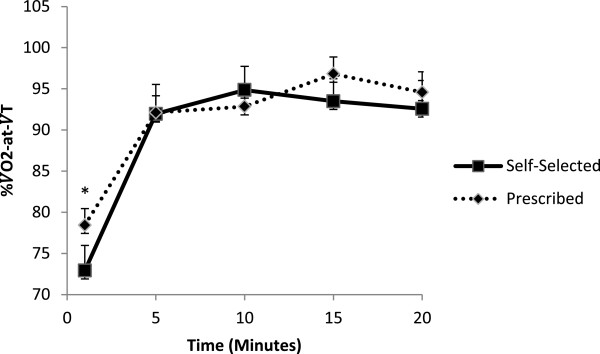
$\%\dot{V}O_{2}‒\mathrm{at}‒\mathrm{VT}$** across time for self-selected and prescribed exercise sessions.** * = significantly lower than all other time points (p < .001), with standard error bars.

A 2 (condition: self-selected or prescribed) by 5 (time: min 1, 5, 10, 15 and 20) repeated measures ANOVA on $\%\pm \dot{V}O_{2}‒\mathrm{at}‒\mathrm{VT}$ showed no significant difference in the intensity participants worked at between the prescribed and self-selected sessions (*F*_(1.00, 26.00)_ = 0.82, *p* = .38, *η*^*2*^_*p*_ = .03) and no time by condition interaction (*F*_(2.8, 74.3 = 1.17)_*p* = .34, *η*^*2*^_*p*_ = .04). Results showed a significant increase in intensity across the 20-minute exercise sessions (*F*_(2.35, 61.07)_ = 28.19, *p* < .001, η^2^_*p*_ = .52) (see Figure 1). Pairwise comparisons revealed that after a significant increase from minute 1 (*M* = 75.67% of $\dot{V}O_{2}‒\mathrm{at}‒\mathrm{VT}$ ± 2.23) to minute 5 (*M* = 92.64 ± 13.74) the exercise intensity remained relatively stable (see Table 2).

**Table 2:** $\%\dot{V}O_{2}‒\mathrm{at}‒\dot{V}T$** across time for self-selected and prescribed exercise session**

**Minute**	**Self-selected session**	**Prescribed session**
1	75.5 ± 12.3*	78.4 ± 10.5*
5	93.8 ± 11.0	92.11 ± 10.7
10	95.8 ± 11.9	92.8 ± 9.9
15	100.2 ± 10.9	96.8 ± 10.7
20	99.7 ± 12.4	94.6 ± 13.0

*% vo2 was significantly lower than all other time points (p < .001).

$\%\dot{V}O_{2}$ peak at minute 1, 5, 10, 15 and 20 of the self-selected exercise session were analyzed to establish the exercise intensity chosen by participants. Results showed that on average participants worked at 72% of $\dot{V}O_{2}\mathrm{peak}\pm 12.99$; within the recommended range from the ACSM of 50-85% $\dot{V}O_{2}$ peak (see Table 3).

**Table 3:** **Descriptive statistics showing change over time in **$\%\dot{V}O_{2}$** peak**

**Time**	**Mean**	**Standard deviation**	**N**
Minute 1	59.58	12.33	27
Minute 5	72.88	11.48	27
Minute 10	74.28	11.74	27
Minute 15	77.43	10.30	27
Minute 20	77.42	10.82	27

### Affective responses

**Figure 2:**
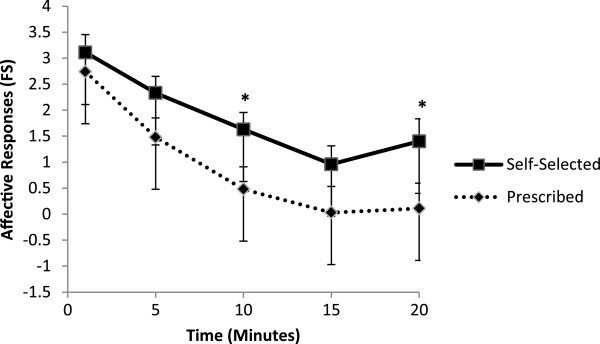
**Affective responses (using the Feeling Scale) across 20-minute exercise sessions, either of self-selected or prescribed intensity.** * = significant differences in affective responses between self-selected and prescribed groups, with standard error bars.

The 3 factor mixed model ANOVA on FS responses found significant main effects for condition (*F*_(1.00, 26.00)_ = 15.87, *p* < .001, *η*^*2*^_*p*_ = .38), and time (*F*_(1.93, 49.74)_ = 41.48, *p* < .001, *η*^*2*^_*p*_ = .62). Participants in the self-selected condition reported significantly more positive affective responses (*M* = 1.89 ± 0.33) than those in the prescribed condition (*M* = 0.97 ± 0.39). FS responses decreased significantly across time, except from minute 15 to 20 (see Figure 2). Results also revealed a significant condition by time interaction (*F*_(2.77, 72.02)_ = 3.03, *p* < .05, *η*^*2*^_*p*_ = .10). Tukey’s post-hoc tests revealed that the condition by time effect occurred as a result of a significant difference in affective responses between self-selected and prescribed conditions at minute 10 and at minute 20. The analyses confirmed that the order of presentation (prescribed or self-selected first) did not influence affective responses, with no significant main effect or interactions involving order.

### Ratings of perceived exertion

A 3 factor mixed model ANOVA on RPE revealed significant main effects for condition (*F*_(1.00, 26.00)_ = 32.2, *p* < .001, *η*^*2*^_*p*_ = .55) and for time (*F*_(1.98, 51.45)_ = 41.88, *p* < .001, *η*^*2*^_*p*_ = .62), but no significant main effect for order or any significant interactions. Results indicated that RPE was significantly higher in the prescribed condition (*M* = 4.87 ± 0.27) than the self-selected condition (*M* = 3.87 ± 0.24). Pairwise comparisons also revealed that RPE scores were significantly lower at minute 1 (*M* = 2.35 ± .29, *p* < .001) and 5 (*M* = 3.96 ± .26, *p* < .001) than all subsequent time points. Results also indicated that RPE responses at minute 10 (*M* = 4.60 ± 0.27) were significantly lower (*p* < .001) than responses at minute 15 (*M* = 5.4 3 ± .31), but no difference was revealed between minute 10 and minute 20 (*M* = 5.48 ± 0.34).

## Discussion

The purpose of this study was to examine the effect that autonomy in the self-selection of exercise intensity has on affective responses and perceived exertion. Although the intensity of both the prescribed and self-selected sessions was the same, and within the ACSM recommended levels, there were significant differences in affective responses and RPE. These results suggest that allowing individuals to self-select exercise intensity elicits a more favourable response to exercise, which may have important implications for encouraging individuals to engage in, and maintain, PA [5,6,9].

Consistent with our hypothesis, affective responses were significantly higher and more positive in the exercise session that was self-selected by the individual compared to affective responses reported in the prescribed session. Importantly, there were no significant differences in exercise intensity between the self-selected and prescribed sessions, suggesting that the differences in affective responses did occur as a result of the nature of the session (i.e., prescribed or self-selected intensity) and were not due to differences in intensity. These findings are consistent with those of Vazou-Ekkekakis and Ekkekakis [8] and suggest that a loss of autonomy over the intensity of an exercise session can have a negative effect on affective responses. However, the current findings differed to those found by Williams and Raynor [24] who did not find a difference in affective response between prescribed and self-selected sessions. This may have been a result of the higher intensity exercise in the current study compared to the intensity of the sessions used by Williams and Raynor, described as ‘leisure’ rather than exercise. Consequently, the current findings may be relevant to an exercise behaviour but not leisure behaviour.

It is important to note that, despite no difference in exercise intensity between the two conditions, RPE were higher when the intensity was prescribed compared to when self-selected. Previous research has demonstrated different perceptual ratings and intensities when the process has varied between effort estimation versus production [25]. When actively regulating (the process involved in a self-selected session) perceptual responses were shown to be lower for a given intensity than when the process was passive.

The current study, coupled with a growing body of research, suggests that by allowing individuals autonomy over their own exercise intensity they will choose to exercise at an intensity that is of a sufficient intensity to benefit health. In the current adolescent population, when participants self-selected their exercise intensity they worked on average at 72 $\%\dot{V}O_{2}\mathrm{peak}\text{,}$ well within the recommended ACSM range. Given the benefits that exercising within this range has been shown to elicit [39-41], it is crucial to identify how individuals can be encouraged to work at this intensity. The current study examined the exercise intensity of self-selected exercise in adolescents, and although a different population from previous studies, results indicated that self-selected exercise intensity was within the recommended range of between 50-85% $\dot{V}O_{2}\mathrm{peak}\text{,}$ supporting previous findings with middle-aged adult participants [13,42,43] and college-age participants [8,18,44,45]. The results from the current study also show that when given the chance to self-select exercise intensity, adolescents chose to modify the intensity throughout the session (particularly in the first 10 minutes), suggesting that allowing changes only every 5 minutes (as per previous studies) is limiting, particularly during the initial stages of exercise when participants are finding a comfortable pace to work at.

### Theoretical implications

Recent evidence has highlighted the importance of affective responses to PA in motivation for future participation [5,6]. Deci and Ryan [22,23] suggested that the degree of pleasure that individuals experience when they act autonomously will be higher than that experienced when behavioral parameters are externally controlled. The current findings regarding the importance of autonomy for the affective responses of adolescent girls to PA are congruent with SDT, and suggest it may be a crucial theoretical framework to help understand PA behavior in this population. Consistent with the present findings, research has shown that prescribed conditions, or those not under one’s autonomy, were associated with less positive affective states compared to autonomous, or self-selected conditions [8,11,12,24,46,47].

### Practical implications

The combination of individuals choosing to work within the recommended range, and the elicitation of more positive affective responses in a self-selected exercise session could contribute to a re-focusing of exercise prescriptions and a move away from prescribed, formalized exercise sessions [14]. The adolescents chose to work at an intensity that was pleasurable, safe and effective, complying with a tripartite approach to exercise prescription [16]. Ekkekakis et al. [16] recognise that the benefit and risk of exercise must be considered in exercise prescription, but argue that the intensity that is more likely to increase pleasure, thus potentially support motivation and adherence, is of equal importance. This may be particular relevance to adolescents, given the decline in PA that is evident as individuals enter adolescence [2,3]. Allowing adolescents to choose the intensity of their exercise session, and giving them autonomy over it, may be a first step in encouraging adolescent females to be more active and promote enjoyable exercise experiences. This is preferable to promoting externally controlled, prescribed sessions that have the potential to elicit more negative affective responses and impact future participation [6,12]. As such, the optimization of affective responses should be taken into account when recommending or prescribing PA to the public. Indeed, results from the current study highlight the importance of considering psychological factors in the prescription of PA guidelines. Specifically, the data provides a strong rationale for the promotion of self-paced PA in order to achieve more positive affective responses.

### Limitations

Whilst this study sought to identify whether autonomy over one’s exercise experience contributed to affective responses a limitation of the current study was that participants were not given autonomy over the duration or modality of their exercise sessions. By allowing individuals to choose their own mode of exercise, autonomy may be further supported, which has the potential to further enhance affective responses and in turn increase adherence to an exercise programme. Future studies should seek to explore this concept further by giving participants choice over modality within the gym, for example choosing between an exercise bike, treadmill or rowing machine, as well as examining whether exercise duration has an effect.

A further potential limitation is that the use of an electric treadmill to simulate a situation of self-selected intensity is problematic. When running on the treadmill, the speed is determined by the machine forcing the body to comply, rather than self-selected as if one was to run outside. Though, treadmills are commonly available tools that are used in exercise settings and this may offset the apparent limitation. Future research should seek to explore whether self-selected exercise in a more naturalistic environment (i.e. running outside) would also elicit the same affective responses.

## Conclusion

Results supported the hypothesis, with affective responses being more positive during the self-selected session than during the prescribed session, and ratings of perceived exertion were lower during the self-selected session than during the prescribed session, despite the matched intensity. With the current decline in physical activity in adolescent females these findings are particularly relevant. To identify ways of enhancing one’s exercise experience with the aim of improving adherence to exercise may be useful in stopping this decline in PA. The current study demonstrated that allowing adolescent females autonomy over the intensity of their exercise session, instead of prescribing it and removing that autonomy, elicited more positive affective responses and lower RPE. These findings demonstrate the importance of autonomy and self-paced PA in the public health domain and support several studies highlighting the relationships that exist between autonomy and self-paced PA and affective response to exercise. Even though the intensity did not differ between the self-selected and prescribed sessions, there was a significant impact on the quality of the affective experience, which may have potential long-term implications for adherence to exercise.

## Abbreviations

PA: Physical activity; RPE: Ratings of perceived exertion; FS: Feeling scale; ACSM: American College of Sports Medicince; SDT: Self determination theory; BMI: Body mass index; E-P: Eston-Parfitt curvilinear ratings of perceived exertion scale; GXT: Graded exercise test.

## Competing interests

The authors declare that they have no competing interests.

## Authors’ contributions

CHW conceived the study and participated in its design and coordination. CHW carried out all data collection and analyses, interpretation of data, and drafted the manuscript. PF was involved in the analysis and interpretation of data, drafting the manuscript and revisions of subsequent drafts providing a critical review. GP was involved in the study design, interpretation of data, drafting of the manuscript and subsequent revisions. All authors read and approved the final manuscript.

## Pre-publication history

The pre-publication history for this paper can be accessed here:

http://www.biomedcentral.com/2052-1847/6/35/prepub
